# A Field Study Into Hong Kong’s Wet Markets: Raised Questions Into the Hygienic Maintenance of Meat Contact Surfaces and the Dissemination of Microorganisms Associated With Nosocomial Infections

**DOI:** 10.3389/fmicb.2019.02618

**Published:** 2019-11-12

**Authors:** Man Ying Lo, Wing Yui Ngan, Shue Man Tsun, Huey-Leng Hsing, Kin Tak Lau, Hing Pui Hung, Si Lok Chan, Yan Yin Lai, Yuan Yao, Yang Pu, Olivier Habimana

**Affiliations:** School of Biological Sciences, The University of Hong Kong, Pok Fu Lam, Hong Kong

**Keywords:** wooden cutting board, surface hygiene, biofilms, resident flora, clinical strains, antibiotic resistance genes

## Abstract

Millions every day purchase their raw meat in wet markets around the globe, especially in Hong Kong city, where modern and a traditional way of living is made possible. While food hygiene standards in Hong Kong have more recently focused on the safety of meat sold in these wet markets, the hygienic surface level of wooden cutting boards used for processing meats is seldom observed. This original study performed microbial community profiling, as well as isolating and identifying various strains multiple wooden cutting boards from nine wet markets located on Hong Kong Island. Our study also investigated the efficiency of scraping the surface of cutting boards as a traditional cleaning technique in Hong Kong. Results indicate that these hygienic practices are inefficient for guarantying proper surface hygiene as some most tested cutting boards were found to harbor microbial species typically associated with hospital nosocomial infections, such as *Klebsiella pneumoni*ae. Further analysis also led to discovering the presence of antibiotic-resistant genes (ARGs) among isolated strains. Our results showcase the significance and effects of cross-contamination in Hong Kong wet markets, especially with regards to the potential spreading of clinically-relevant strains and ARGs on food processing surfaces. This study should, therefore, serve as a basis to review current hygienic practices in Hong Kong’s wet market on a larger scale, thereby improving food safety and ultimately, public health.

## Introduction

Hong Kong’s wet markets are known to supply fresh produce and raw meat products to millions of people every day. While safety standards have placed much emphasis on the safety and quality of processed fresh meat in these wet markets (Otoole, 1995; Wu et al., 2014), the hygienic level of wooden cutting boards used for processing these meats is seldom observed, if not entirely ignored. Moreover, although the use of wooden cutting boards for food processing has been regarded as being more hygienic than plastic due to their reported advantageous surface topology or antimicrobial characteristics (Aviat et al., 2016), their lack of maintenance and poor sanitary practices might still outweigh their reported positive attributes, by increasing the propagation risk of relevant foodborne pathogens (Edelmeyer, 1984).

Even though existing global food safety norms help improve and standardize manufacturing practices, good hygienic practice, hazard analysis critical control point, for safer processed foods (Mahajan et al., 2014), these may not necessarily be implemented in traditional wet markets around the world, thereby causing food safety concerns (Blanck et al., 2014). In the case of Hong Kong, a hygienic practice traditionally embraced by local stall keepers generally involve water rinsing and physical scraping off the cutting board’s surface layer. Elsewhere in Africa, few reports described the cleaning of utensils or cutting from inadequate wet markets facilities (Fasanmi et al., 2010; Aburi, 2012; Chepkemoi et al., 2015). In Greece, the interrelationships between hygienic conditions during meat processing and personal hygiene were revealed to be primary factors affecting meat quality and shelf life in retail outlets (Andritsos et al., 2012). Ensuring cutting board hygiene should, therefore, be considered when implementing proper hygienic practices, since the use of unhygienic cutting boards may become a dangerous source of transferable foodborne disease-causing organisms (Carpentier, 1997).

Moreover, the presence of high nutrient availability in combination with high humidity, affecting the moisture content of wood, may also play an essential role in the proliferation and survival of microorganisms on irregular and porous wooden cutting board surfaces (Carpentier, 1997). The maintenance and hygiene monitoring of wooden food-contact surfaces should therefore serve as a practical means to reduce the level of cross-contamination (Aviat et al., 2016), by ensuring regular cleaning and sanitation, which are essential practical measures for ensuring the microbiological load of wooden food contact surfaces (Kim et al., 2012; Falco et al., 2019). While the World Health Organization has recommended that governments prioritize food safety and public health throughout the food chain, hygienic regulations on the matter of wooden food contact surfaces differ from one global region to another, with countries even lacking clear defined regulations (Aviat et al., 2016). Interestingly, a survey conducted by the Food and Drug Administration has revealed that high-risk practices of handlers were found to be the principal cause for cross-contamination from poorly maintained wooden cutting boards (Klontz et al., 1995).

The level of food safety knowledge among market vendors is also an essential factor for implementing proper hygienic practices (Mahajan et al., 2014). In one study, it was shown that many Asian food peddlers were not adequately trained on the issue of public health management or food safety standards for avoiding the spread of foodborne illnesses (Minh, 2017; Ghatak and Chatterjee, 2018; Tran et al., 2018). Adding to Hong Kong’s high ambient temperatures and customer densities within markets, conditions favoring the growth, colonization and spread of food-borne pathogens on these cutting boards can be enhanced (Kotzekidou, 2016).

The lack of chopping board hygiene or their improper maintenance may, therefore, lead to the establishment of biofilms niches within the cutting board surface (Edelmeyer, 1984; Prechter et al., 2002). The presence of such biofilms is known to contribute to favor the unwanted establishment of pathogens and the spread thereof into other environments. Biofilms are formed by a complex community of microorganisms on both biotic or abiotic surfaces, producing extracellular polymeric substances (EPS) that allow the survival of microorganisms in harsh environments, as well as under antimicrobial attacks (Flemming and Leis, 2001; Stewart, 2001, 2003). The potential threat of such biofilms present on cuttings boards in Hong Kong wet markets has not been thoroughly investigated, nor has the hygienic efficiency of the traditional scraping fully elucidated. While scraping is considered as an ideal destructive means to recovering microorganisms on types of surfaces with the advantage of extracting organisms at different surface depths (Ismaïl et al., 2013), its usage for ensuring surface hygiene for processing meat remains questionable. Part of the question lies with the reliability of the microbiological load on the newly exposed surface following scraping, combined with the level of top layer removal by the scraping operator. It is, therefore, within these contexts that this study aimed at identifying and isolating species isolated from various wood cutting boards from wet markets located on Hong Kong Island. Our second objective was to study the efficiency of scraping the surface of cutting boards as a traditional cleaning technique. These aims were achieved by analyzing swab samples from different wet market stalls and samples following various cleaning procedures on a recently used wet-market cutting board, by applying a combination of traditional microbiological techniques, sequencing and data analysis as well as advanced high-resolution microscopy.

## Materials and Methods

### Study Area

A total of 15 wooden cutting board swabbing samples were obtained from nine wet-markets in Hong Kong Island, including Ap Lei Chau Market (AL), North Point Market (NP), Sai Ying Pun Market (SY and CSF), Shek Tong Tsui Market (ST), Sheung Wan Market (SW), Smithfield Market (SF) and Wan Chai Market (WC and TW). Sampling sites were selected based on their proximity relative to the University of Hong Kong for facilitating the processing of swab samples.

Random short surveys were also conducted to assess common cleaning methods typically employed for cleaning butcher block countertops in wet markets, as well as their frequency. Interviews were carried out in four local pork stores from ST and SF.

### Swab Sampling, Culturing, and Isolating

**FIGURE 1:**
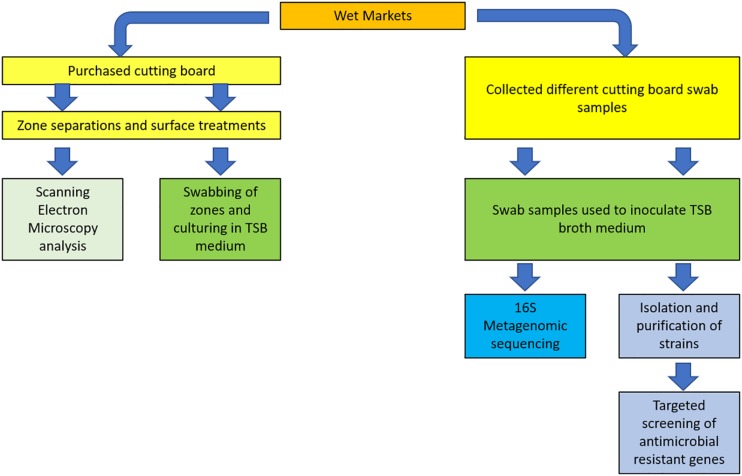
Schematized summary of study design.

Swab sampling was performed on butcher countertops, from which pork meat processing was normally performed. Based on the review by Ismaïl et al. (2013), the recovery of bacteria using a non-destructive method such as swabbing, was described as not being suitable on irregular surfaces, as opposed to the friction method, which could be applied on rougher surfaces. Considering the rough, porous nature of wooden cutting boards (Aviat et al., 2016), we hence used swab tip to scrub on cutting board surfaces, which was found to be a practical, non-destructive, non-invasive means for standardizing our sampling during this study. The microorganism recovery method implemented in this study, therefore, applied a combination of both swabbing and friction. For the sake of simplifying the description of the method, the term “swabbing” is henceforth used in this report. Briefly, pre-sterilized cotton swabs were hydrated with sterile PBS before swabbing a surface area equivalent to 10 cm^2^ on the most active area of the cutting boards. The sampled swab was then placed in tubes containing sterile 2 mL Amies Charcoal transport medium. Tubes were subsequently placed in ice buckets during transportation to HKU for further processing. For bacterial isolation, swabbed samples were first cultured in 100 mL Bacto^TM^ Tryptic Soy Broth (TSB) medium (BD^TM^, United States), which were then incubated at 30°C for 20 h. The overnight culture was then sub-cultured by spread plating onto either Difco^TM^ Tryptic Soy Agar (TSA) (BD^TM^, United States) and Difco^TM^ Violet Red Bile Agar (VRBA) (BD^TM^, United States), and incubated at 30°C for 20 h. Selected isolates were inoculated in TSB and incubated at 30°C for 20 h. Pure cultures were mixed with sterile 40% glycerol solution at a 1:1 ratio before storage at −80°C. A summary of the various steps described in the following section is illustrated in the schematized study design (Figure 1).

#### Metagenomic Profiling of Environmental Swab Samples and Isolate Identification

Genomic DNA was extracted for metagenomic amplicon sequencing from 15 overnight culture samples described in section “Swab Sampling, Culturing, and Isolating.” For isolate identification, DNA extraction was performed from monocultures of reactivated pure isolates cultured in TSB at 30°C for 20 h. Genomic DNA extraction procedure was performed using PureLink Microbiome DNA Purification Kit (Invitrogen^TM^, United States) A29790 according to the manufacturer’s protocol. The concentration and purity of extracted DNA were measured with BioDrop DUO (BioDrop, United Kingdom). For 16S metagenomic analyses, genomic DNA was sent to Novogene (Shenzhen) for PCR-free library preparation and Illumina HiSeq PE250 sequencing of 16S V3-V4. Metagenomic analyses were then analyzed using Parallel-Meta pipeline (Jing et al., 2017). The generated raw sequence data were deposited at the European Nucleotide Archive, accession number PRJEB33545.

The identities of isolate pure cultures were confirmed following PCR implication of the 16S rRNA marker followed by Sanger sequencing using Applied Biosystems (ABI) 3730xl DNA Analyzer (Centre for Genomic Sciences, Li Ka Shing Faculty of Medicine, HKU). The 16S reverse and forward primers are listed in Table 1. A nucleotide BLAST DNA search was then performed with DNA database software of the NIH (United States) from the amplicon sequences obtained for the identification of isolates in the website: http://www.ncbi.nlm. nih.gov.

**TABLE 1:** Reverse and forward primers for the amplification of the 16S rRNA gene and a selection of representative antibiotic resistance genes used in this study.

**Gene product**	**Primer sequence (5′→ 3′)**	**References**
Bacteria Universal	27F: AGAGTTTGATCMTGGCTCAG	Weisburg et al., 1991; Stackebrandt and Liesack, 1993
	1492R: GGTTACCTTGTTACGACTT	
*tet*(*M*)	F: GCTTATTCCGGGGAAATTGT	Szczepanowski et al., 2009
	R: CGGGTCACTGTCGGAGATT	
*Bla*TEM	F: GTGCGCGGAACCCCTATT	de Paula et al., 2018
	R: TTACCAATGCTTAATCAGTGAGGC	
*erm*(*B*)	F: AGCCATGCGTCTGACATCTA	Szczepanowski et al., 2009
	R: CTGTGGTATGGCGGGTAAGT	
*qnr*(*S*)	F: GATATCGAAGGCTGCCACTT	Rutgersson et al., 2014
	R: CACGGAACTCTATACCGTAGCA	
*mex*(*B*)	F: GACCAAGGCGGTGAAGAAC	Szczepanowski et al., 2009
	R: AACACCTGGAAGTCACCGAC	

#### Identification of Antibiotic Resistance Genes (ARGs) From Isolated Strains

Five oligonucleotide primers were designed for the amplification of representative antibiotic resistance genes in selected identified isolates. PCR runs required a 15 μL of the reaction mixture, including 14 μL of the isolate DNA and 1 μL of selected primers. Each isolate DNA have been prepared with five different types of primers separately in a 96-well plate. The antibiotics resistance genes were screened by sanger sequencing at the Centre for Genomic Sciences. All primer reagents were supplied by Tech Dragon Limited, Hong Kong (Table 1).

Validation of obtained sequences was checked by performing BlastX at NCBI DNA database software mentioned in section “Metagenomic Profiling of Environmental Swab Samples and Isolate Identification” to assess the identity of translated proteins from amplified sequences.

### Cutting Board Sample Processing

One pre-owned butcher cutting board was purchased from Shek Tong Tsui Market for testing the impact of cleaning strategies on wooden food contact surfaces. In this instance, the commonly used surface scraping technique was compared to a Cleaning-In-Place (CIP) strategy (Bremer et al., 2006). Given the limited size of the cutting board, three random areas of 16 cm × 3.5 cm were delimitated with the help of a prepared plastic film frame. In each selected area, three zones of 4 cm × 2.5 cm were demarcated for processing.

Control zones were processed by wetting surface using 400 μL sterile phosphate-buffered saline (PBS) solution, followed by swabbing the area with sterile cotton swabs and spreading on to TSA plates. Selected areas were swabbed a second time by a new cotton swab and processed as previously described in section “Swab Sampling, Culturing, and Isolating.”

The scraping cleaning method was performed on a second zone by first scraping delimitated area by knife until a whiter layer was distinctly removed. The new surface was then wetted and swabbed as previously described for the control zone.

To test the effectiveness of different concentration of reagent, 5.2% sodium hypochlorite solution, had been diluted to 0.1 and 0.024% of sodium hypochlorite solution (Agriculture and Food Standards Policy Committee, 1991). The area was first cleaned by 400 μL 0.024% sodium hypochlorite solution with two cotton swabs for 5 min and waited for 20 min before sampling. Sample collecting procedures and plate spreading on TSA were conducted as previously described for control zones. In the CIP treatment zones, an additional CIP treatment was performed using 0.1% sodium hypochlorite solution from which swab samples were collected and processed as previously described.

#### Microscopic View of the Treated Wooden Board

Three out of four cutout pieces of the butcher block were used to repeat the cleaning strategies described in section “Cutting Board Sample Processing.” Following treatment, the pieces underwent chemical fixation using 10% formaldehyde followed by a dehydration step using a critical point dryer, and gold sputtering before Scanning Electron Microscopy (Hitachi 3400 and S-4800) (Electron Microscope Unit, The University of Hong Kong). Care was taken to score each sample pieces to recognize the chopping board surface during microscopy.

## Results and Discussion

### Metagenomic Profiling of Wet Market Cutting Boards

**FIGURE 2:**
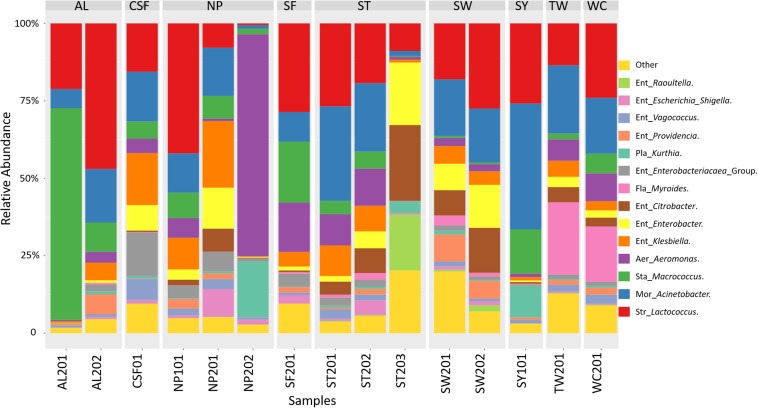
Taxonomic community profiles at the genus level following 16S metagenomic sequencing performed on swab samples collected at nine wet markets in Hong Kong Island, and analyzed using Parallel-Meta.

The analysis of the 16S metagenomic amplicon sequencing data of samples collected from nine wet markets revealed that the cutting boards were principally constituted of microorganisms belonging to the *Proteobacteria*, *Firmicutes*, and *Bacteroidetes* phyla. As presented in Figure 2, the *Proteobacteria* group were composed of genera belonging to *Enterobacteriaceae*, *Aeromonas*, and *Moraxellaceae*. The *Firmicutes* group was made-up of *Staphylococcus*, *Streptococcus*, and *Planococcaceae*, while *Flavobacteriaceae* genus represented the *Bacteroidetes* phyla. Further taxonomic abundance profile analysis between cutting boards revealed that the *Proteobacteria* phylum was composed of genera considered potentially pathogenic such as *Enterobacteriaceae*, *Escherichia*, and *Shigella, or Ent. Klebsiella*, the latter associated with clinical infection (Moremi et al., 2016). Among those representing the *Firmicutes* phyla, the *Planococcaceae* and *Kurthia* genus may also be considered pathogenic, considering known species such as *Kurthia gibsonii* considered as a sexually transmitted zoonosis (Kövesdi et al., 2016). The abundance of these potential pathogenic genera show variations between cutting board samples or wet markets, exemplified by the taxonomic profile of sample NP201 and CSF01 with their highest abundance of *Enterobacteriaceae Escherichia Shigella, or Ent. Klebsiella* compared to other samples. Cutting boards sampled within the same wet market also showed differential abundance profiles, such as samples ST201, ST202, and ST203, the latter sample having a unique profile with a higher abundance of *Ent. Citrobacter*, *Ent. Enterobacter*, and *Staphylococcus Macrococcus* genera. These variations may reflect the individual hygienic state of each cutting board, and hence, the stall keepers own hygienic practices during meat processing, as discussed later in this report. The surprising presence of clinically relevant genera on these cutting board is an alarming finding and one that should question the level of food safety and public health. It is for this reason that an attempt was made to identify species from isolated strains to assess whether the sampled cutting boards were indeed harboring potentially pathogenic organisms.

### Identification of Randomly Isolated Strains

**FIGURE 3:**
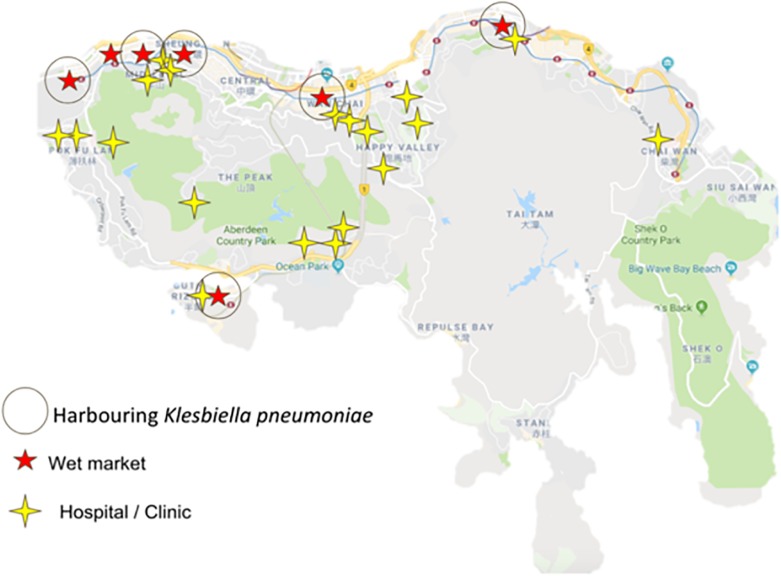
Marked locations of sampled wet markets harboring *Klebsiella pneumonia* and hospitals in Hong Kong Island.

For identification purposes, 60 random colonies were first selected based on their morphologies then post-cultured into TSB. Following overnight culture, their gDNA was extracted for further 16S sequencing (cf. section “Materials and Methods”). Of the 60 isolates (cf. Supplementary Video S1), 16 species were identified as *Aeromonas caviae, Aeromonas hydrophila, Citrobacter freundii, Cronobacter sakazakii, Enterobacter hormaechei*, *Enterococcus faecium, Escherichia coli, Hafnia paralvei, Klebsiella aerogenes, Klebsiella pneumoniae*, *Klebsiella variicola, Kurthia* sp., *Lactococcus garvieae, Proteus vulgaris, Providencia alcalifaciens, Providencia stuartii*, and *Serratia marcescens*. From the illustrated summary in Figure 3, it can be noticed that most sampled area share common species; among the most recurrent are *Klebsiella pneumonia* and *Escherichia coli* which were isolated in six of the nine wet markets. While a more detailed epidemiological study would be needed to ascertain whether recurrent isolated species originate from a specific strain or source, the underlying question that needs to be asked is how non-food related organisms could be sampled and isolated from food contact surfaces. Of note, *Klebsiella* sp., a typical nosocomial species, is known to be an opportunistic pathogen linked to nosocomial infections in immunocompromised individuals (Podschun and Ullmann, 1998). Other isolated strains of the Enterobacteriaceae family such as *Escherichia coli, Proteus* sp. and *Serratia marcescens* are also considered as hospital-acquired infectious agents (Khan et al., 2015). Previous findings from Podschun and Ullmann also showed that *Klebsiella pneumoniae* is typically associated with hospital environments, when comparing stools hospitalized and non-hospitalized patients. The presence of clinically associated organisms on food contact surfaces in this study suggests that wooden cutting boards in Hong Kong’s wet markets are prone to cross-contamination, albeit the exact vector responsible for the contamination of *Klebsiella pneumoniae* cannot be fully elucidated.

**FIGURE 4:**
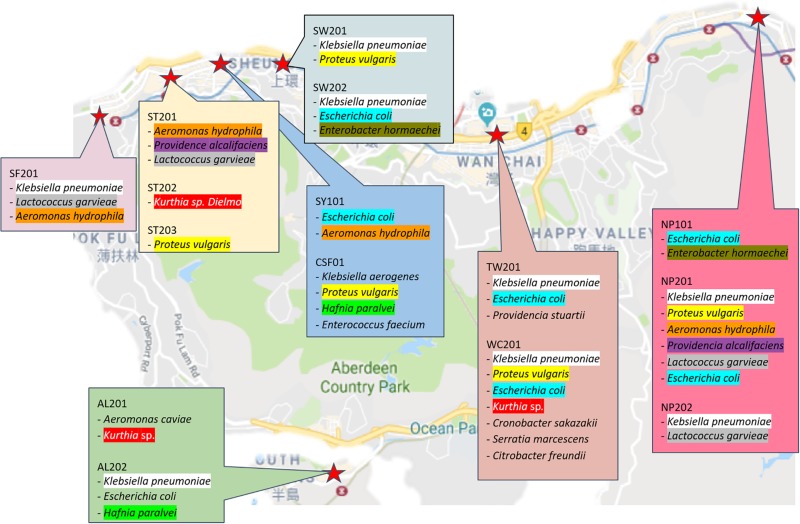
Origin of isolated strains from previously sampled wet markets in Hong Kong Island.

Interestingly, the cuttings boards from which *Klebsiella pneumoniae* strains were isolated are situated in wet markets near hospitals, including general out-patients clinics, as illustrated in Figure 4. Further studies are therefore needed to determine whether isolated strains in this study are linked to strains endemic to hospital environments. With the sheer number of people purchasing their foods in Hong Kong Wet markets, coupled with the combined proximity of clinics/hospitals, the likelihood of cross-contamination through human vectors cannot be discounted (Khan et al., 2015, 2017).

In a recent non-food related study, it was revealed that inner-city traffic flows of the metro system could also contribute to the spread and exposures of microbiome and resistome in the environment (Kang et al., 2018). The flow and relocation of bacterial colonies were examined across different lines in the Hong Kong Mass Transit Railway system among the morning and afternoon. Their results suggested that morning microbiome originating from the busiest traffic line was also isolated on other interchangeable lines later in the afternoon.

Likewise, while some of the wet markets were not necessarily located in the proximity of hospitals or clinics, clinically relevant organisms may still have been transferred to easily accessible food processing areas, given enough flows of traffic and people during market hours.

### Targeted Antibiotic Resistance Gene-Screening From Isolated Strains

Having established the possibility of the spread of clinically relevant organisms onto food contact surfaces, the question of whether antibiotic resistance genes can likewise be spread in such food processing environments is also meaningful. Here, all 60 isolates were screened against selected classes of targeted antibiotic resistance genes as described in section “Identification of Antibiotic Resistance Genes (ARGs) From Isolated Strains.” Results from this screening presented in Table 2 revealed that only five wet market isolates tested positive for only three antibiotics resistance genes *tet*(*M*), *BlaTEM*, and *mex*(*B*). Three *Lactococcus garvieae* isolated from two wet markets tested positive for *mex*(*B*), a multidrug efflux gene against various antibiotics (Szczepanowski et al., 2009). Moreover, *Lactococcus garvieae* can be regarded as a low virulence organism, rarely associated with human infections associated with bacteremia and Urinary Tract Infections (Choksi and Dadani, 2017). *L. garvieae* is well known as a fish pathogen (Navas et al., 2013), and its presence on cutting boards meant for processing beef or pork is reflective of cross-contamination, especially if the cutting boards had a history of contact with raw fish. The positive presence of *mex*(*B*) gene also suggests that isolated *L. garvieae* strains may be of clinical origin, having been regularly exposed to a wide range of antibiotic stressed environments.

**TABLE 2:** Isolates that tested positive for harboring antibiotic resistance gene(s).

	**Sample code**	**Presumed identification**	***tet*(*M*)**	***Bla*TEM**	***mex*(*B*)**
1	NP201_T5	*Lactococcus garvieae* strain JB377100 16S ribosomal RNA gene, partial sequence			+
2	NP201_V4	*Escherichia coli* strain WCHEC4533 chromosome, complete genome	+	+	
3	NP202_T2	*Lactococcus garvieae* DNA, complete genome, strain: 122061			+
4	SF201_T5_1	*Lactococcus garvieae* strain JZ R-138 16S ribosomal RNA gene, partial sequence			+
5	ST203_T3	*Proteus vulgaris* strain FDAARGOS_366 chromosome, complete genome			+

One presumed *Proteus vulgaris* strain was also found to harbor the *mex*(*B*) gene. *Proteus vulgaris* is typically endemic the intestines of human and animals, however, it can be isolated from individuals in long-term care facilities and hospitals and patients with underlying diseases or compromised immune systems. Treatment of *Proteus vulgaris* usually involves the use of antibiotics, thereby explaining the organisms’ efflux pump resistance mechanism. Efflux pumps originally played the role of extruding toxic substances out of cells, however, with several exposures to antibiotics an over-expression of efflux pump genes may help the cells to increase their survival under antibiotic-stressed environments (Webber and Piddock, 2003). The likely source of this *Proteus vulgaris* strain cannot be determined since antibiotics are typically used in both animal husbandry and hospitals. Further studies will be needed to determine whether *Proteus vulgaris* had been linked to previous clinical infections.

One isolated *Escherichia coli* strain was found to be positive for both *tet*(*M*) and *BlaTEM* genes. Considered as an indicator of poor surface hygiene, the presence of *Escherichia coli* with antibiotic multi-resistance capabilities on meat cutting boards should be of public health concern. The production β-lactamases – TEM in *Escherichia coli* was shown caused about 90% of antibiotic ampicillin resistance (Cooksey et al., 1990). Some of its harmful strains could also resist to antibiotics of sulfonamides, streptomycin and apramycin, whereas only in the strains of enterotoxigenic *E. coli* was found to resist to quinolones (Boerlin et al., 2005). Interestingly, β-lactamases – TEM is a plasmid-encoded enzyme that provided bacteria with multi-resistance against β-lactam antibiotics (Emery and Weymouth, 1997), which suggests that multi-resistance genes may be transferred for one organism to another, especially within the context of microbial biofilms, where DNA and other materials are easily interchanged between microorganisms (Anjum and Krakat, 2016).

### Surface and Hygienic Assessment of a Wooden Cutting Board

The combined presence of potential clinically associated organisms and ARGs led to question the hygienic routines implemented in the Hong Kong wet markets. Poor hygiene on surfaces such as cutting boards may lead to the formation of biofilms that can potentially allow the proliferation of unwanted organisms and the spread of ARGs. Observations of daily routines of wet market meat stalls revealed that stall keepers would typically scrape off the top surface of their cutting boards using a knife. Further enquiry into the scraping method by impromptu interviewing of stall keeper revealed that it is the most commonly adopted cleaning practice. Based on observations of wet market practices in this study, cutting boards are usually scraped until a white layered film is visibly removed from the cutting area, before processing meat. Among those interviewed, only one stall keeper used domestic dishwashing detergent and mechanical brushing as a means to ensure cutting board surface hygiene. Regardless of the method employed, little is known of how often these surface hygiene routines are carried out, or whether these are implemented between meat-batches. Proper surveying of wet-market hygienic habits would, therefore, help develop best-practice means to ensure proper cutting boards in meat stalls in the longer run. Unfortunately, there are no existing clear rules or guidelines aimed explicitly toward improving the maintenance of wooden cutting boards in Hong Kong’s wet markets. According to the Hong Kong Government’s Food and Environment Hygiene Department own Food Hygiene Code, food contact surfaces of equipment used for processing foods should (A) consist of a non-toxic, non-absorbent, non-corrosive, smooth material; (B) be unaffected to grease, food particles or water; (C) be free from cracks, crevices, open seams; (D) be effectively cleaned, sanitized and; (E) be easily accessible for cleaning, sanitizing and inspection (Anonymous, 2018). Based on these guidelines alone, the systematic use of wooden chopping boards for processing meat in Hong Kong’s wet markets, being a highly absorbent and highly irregular, non-smooth food contact surface should be further examined.

Moreover, the Food Hygiene Code further formulated a rationale stipulating that food contact surfaces should not introduce into food any substance which may be harmful to the health of consumers, however, fails to mention the potential cross-contamination specifically and spread of pathogenic microorganisms. Nevertheless, effective cleaning and sanitizing, facilitated by properly designed food contact surfaces, was identified as an essential driver for ensuring proper surface hygiene. However, wooden chopping board was demonstrated as being an unsuitable material, based on inefficient cleaning and sanitizing outcomes.

**FIGURE 5:**
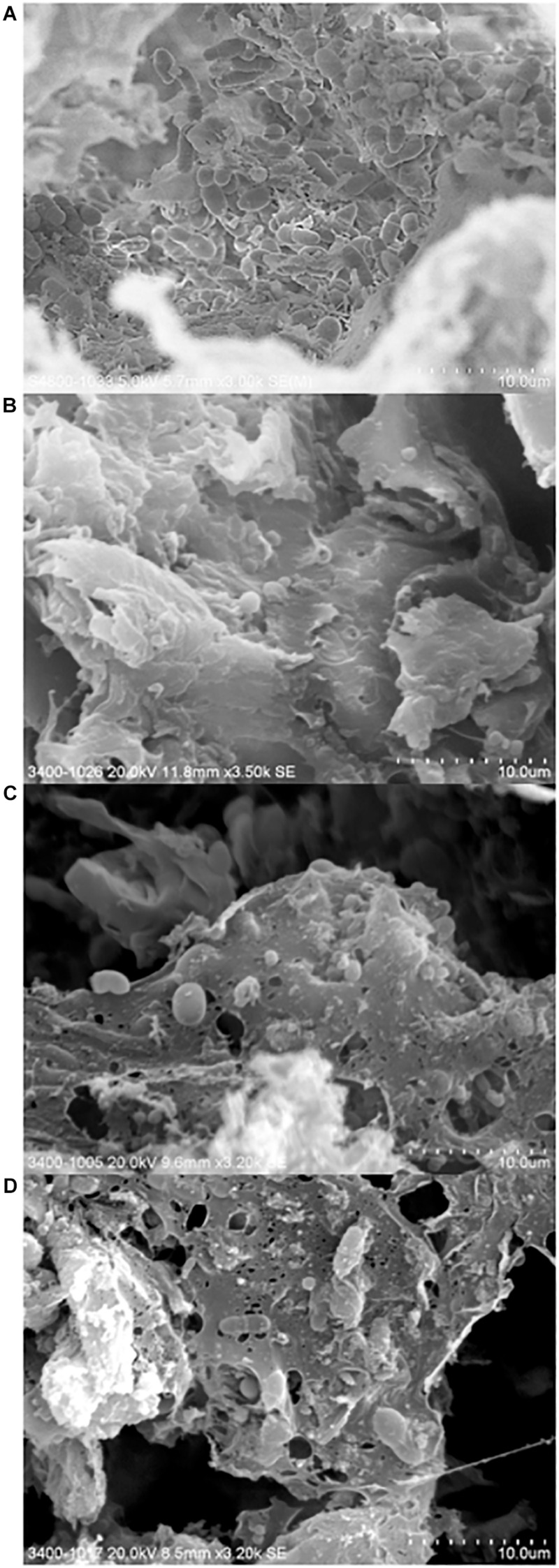
Scanning electron micrographs of chopping board surface that underwent no hygienic treatment **(A)**, traditional scraping **(B)**, 0.024% sodium hypochlorite treatment **(C)**, and 0.1% sodium hypochlorite treatment **(D)**.

Here, we investigated the surface of a purchased cutting board using high resolution scanning electron microscopy following different hygienic treatments (Figure 5). Microorganisms were found in abundance and diversity in all cutting board samples, including those that underwent traditional cleaning and sodium hypocrite treatments. Interestingly, rod- and cocci-shaped bacterial cells as well as budding yeast cells, could be observed in all samples. In a recent Indian market place study, the presence of yeast and fungi on cutting board was suggested to have originated from meat (Thanigaivel and Anandhan, 2015).

The presence of what can be described as remnants of film-like extracellular polymeric substances were also observed in some of the samples, thus suggesting that the microbial cells grow and survive on cutting board surfaces by adopting a strategic biofilm mode of life. Conditions at the surface cutting board may be considered as ideal for biofilm formation, not only because of its unregular topography (i.e., non-smooth surface), but also of extrinsic factors favorizing microbial life such as the surrounding environments (i.e., Hong Kong’s ambient temperature and high humidity), and the constant supply of nutrients from meat pieces. The presence of biofilms within the inner layers of the cutting board may provide embedded organisms protection against being “scraped off” during traditional cleaning of cutting boards, as well as provide a shield against sanitizing agents. The presence of biofilms may also increase the risk of horizontal gene transfer (Hausner and Wuertz, 1999; Sorensen et al., 2005), possibly transferring and further spreading ARGs to commensal or environmental strains. The microbial consortium of bacterial and eukaryotic cells observed on the cutting board points to the need to further our understanding of mixed-species biofilms in the context of harboring pathogenic organisms and their associated resistance profiles to antimicrobials.

**FIGURE 6:**
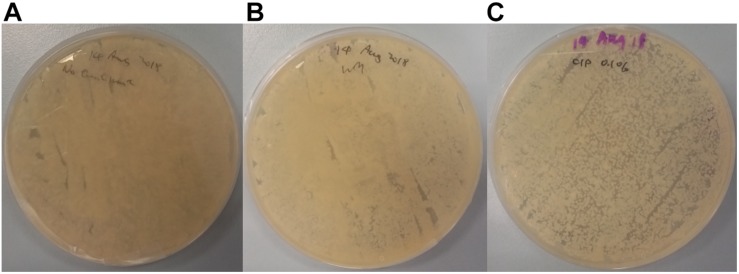
TSA plate spread from swab samples obtained from cutting board surfaces that underwent no hygienic treatment **(A)**, wet market traditional scraping **(B)**, and 0.1% sodium hypochlorite treatment **(C)**.

Further analysis of plated swab samples following cutting board surface treatments (Figure 6) revealed that food contact surfaces remain laden with microorganisms, especially after implementing the traditional scraping technique. Compared to the non-treated control, no differences in growth outcomes on TSA were observed from plated swab samples from scrapped surfaces. Although, surfaces treated using CIP strategy did, however, lead to lower bacterial load on TSA plates compared to other plated swab samples; the presence of such bacterial growth following treatments is an indication of the challenges faced with ensuring surface hygiene on wooden cutting boards in Hong Kong wet markets and highlights the risk of cross-contamination. A former study conducted by Gehrig et al. (2000) showed that high humidity environments could lead to the persistence of spiked *Escherichia coli* cells on wooden cutting boards. However, it was shown that a significant decrease of bacteria count could be achieved on wooden cutting boards when manually brushing and washing with detergent, followed by rinsing under warm water. Although their obtained result seems promising, one should still consider the presence of complex microbial consortia, and the likely presence of biofilms that can interfere with the hygienic maintenance on wet market cutting boards. Nevertheless, only one of all interviewed stall keepers described the use of brushing and dish detergent in their cutting board hygienic maintenance routine.

Top layer scraping of chopping boards is not a suitable means to ensure food contact surface hygiene. Although scraping may be considered as an ideal monitoring method for extracting bacteria from surfaces (Ismaïl et al., 2013), its use as a traditional hygienic benchmark should be questioned. The underlying issue with scraping is the level of depth at which the top layer is removed, which also may or may not be influenced by the technique or the handler (cf. Supplementary Video S1), as well as the initial microbiological load before scraping. Moreover, the knife used during scraping may also be the same knife used during meat processing, in which case, may transfer microorganisms onto the freshly exposed contact surface. Future studies should, therefore, investigate and characterize the hygienic practices in Hong Kong wet markets, together with an extensive survey of cross-contamination risks. Whether other food contact surface material may be used as a suitable alternative for meat processing should also be investigated, not only on matters about efficient cleaning and sanitation but also on their propensity to cross-contamination within Hong Kong’s wet market. On aspects concerning microbial ecology on wet market cutting boards, the survival of clinically relevant organisms, such as *Klebsiella pneumoniae* associated with multi-species biofilm consortia, as well as the transfer and spread of ARGs within such biofilms, should also be explored.

## Conclusion

In this study, microbial profiling was performed on cutting boards used for processing meat in Hong Kong Island wet markets, revealing the alarming presence of clinically relevant microorganisms and the prevalence of antibiotic resistance genes. While the exact transmission routes cannot be concluded from this study alone, obtained results can nevertheless help create awareness of the significance of cross-contamination in Hong Kong wet markets, especially with regards to spreading clinically-relevant strains and ARGs to other areas. This study should, therefore, serve as a basis to review current hygienic practices in wet markets in Hong Kong and globally on a larger scale, thereby improving food safety and ultimately, public health.

## Data Availability Statement

The generated raw sequence data were deposited at the European Nucleotide Archive, accession number PRJEB33545.

## Author Contributions

ML, WN, ST, KL, HH, H-LH, SC, YL, and YP collected the data. All authors analyzed and interpreted the data. OH conceived the conception or design of the work. ML, WN, and  OH drafted and revised the manuscript.

## Conflict of Interest

The authors declare that the research was conducted in the absence of any commercial or financial relationships that could be construed as a potential conflict of interest.

## References

[B1] Aburi P. A. S. (2012). *Assessment of Hygiene Practices Used by Small Butchers and Slaughter Slabs in Beef Value Chain in Juba Town-South Sudan.* Wageningen: Wageningen University and Research.

[B2] Agriculture and Food Standards Policy Committee. (1991). *Code of Practice for Equipment and Procedures for The Cleaning and Disinfecting of Milking Machine Installations (BS 5226:1991).* United Kingdom: British Standards Institution.

[B3] Andritsos N. D. Mataragas M. Mavrou E. Stamatiou A. Drosinos E. H. (2012). The microbiological condition of minced pork prepared at retail stores in Athens, Greece. *Meat Sci.* 91 486–489. 10.1016/j.meatsci.2012.02.036 22459497

[B4] Anjum R. Krakat N. (2016). Detection of multiple resistances, biofilm formation and conjugative transfer of *Bacillus cereus* from contaminated soils. *Curr. Microbiol.* 72 321–328. 10.1007/s00284-015-0952-1 26650381

[B5] Anonymous (2018). *Food Hygiene Code.* Available at: https://www.fehd.gov.hk/english/publications/code/allc_2.html (accessed September 20, 2018).

[B6] Aviat F. Gerhards C. Rodriguez-Jerez J. J. Michel V. Le Bayon I. Ismail R. (2016). Microbial safety of wood in contact with food: a review. *Compr. Rev. Food Sci. Food Saf.* 15 491–505. 10.1111/1541-4337.12199 33401823

[B7] Blanck M. Mirulla R. Rosales M. (2014). “The way forward for better food safety and nutrition: an online discussion,” in *Street Food: Culture, Economy, Health and Governance*, eds Vieira Cardoso R. D. C. Companion M. Marras S. R. (Abingdon: Routledge), 269–274.

[B8] Boerlin P. Travis R. Gyles C. L. Reid-Smith R. Janecko N. Lim H. (2005). Antimicrobial resistance and virulence genes of *Escherichia coli* isolates from swine in Ontario. *Appl. Environ. Microbiol.* 71 6753–6761. 10.1128/aem.71.11.6753-6761.2005 16269706 PMC1287655

[B9] Bremer P. J. Fillery S. Mcquillan A. J. (2006). Laboratory scale Clean-In-Place (CIP) studies on the effectiveness of different caustic and acid wash steps on the removal of dairy biofilms. *Int. J. Food Microbiol.* 106 254–262. 10.1016/j.ijfoodmicro.2005.07.004 16216371

[B10] Carpentier B. (1997). Sanitary quality of meat chopping board surfaces: a bibliographical study. *Food Microbiol.* 14 31–37. 10.1006/fmic.1996.0061

[B11] Chepkemoi S. Lamuka P. O. Abong’ G. O. Matofari J. (2015). Sanitation and hygiene meat handling practices in small and medium enterprise butcheries in Kenya-case study of Nairobi and Isiolo Counties. *Int. J. Food Saf.* 17 64–67.

[B12] Choksi T. T. Dadani F. (2017). Reviewing the emergence of lactococcus garvieae: a case of catheter associated urinary tract infection caused by *Lactococcus garvieae* and *Escherichia coli* coinfection. *Case Rep. Infect. Dis* 2017:5921865. 10.1155/2017/5921865 28811943 PMC5546070

[B13] Cooksey R. Swenson J. Clark N. Gay E. Thornsberry C. (1990). Patterns and mechanisms of beta-lactam resistance among isolates of *Escherichia-coli* from hospitals in the United States. *Antimicrob. Agents Chemother.* 34 739–745. 10.1128/aac.34.5.739 2193616 PMC171683

[B14] de Paula A. C. L. Medeiros J. D. De Azevedo A. C. Chagas J. M. D. Da Silva V. L. Diniz C. G. (2018). Antibiotic resistance genetic markers and integrons in white soft cheese: aspects of clinical resistome and potentiality of horizontal gene transfer. *Genes* 9:E106. 10.3390/genes9020106 29463055 PMC5852602

[B15] Edelmeyer H. (1984). Clean cutting boards and knives is this too much to ask of hygiene. *Fleischwirtschaft* 64 687–690.

[B16] Emery C. L. Weymouth L. A. (1997). Detection and clinical significance of extended-spectrum beta-lactamases in a tertiary-care medical center. *J. Clin. Microbiol.* 35 2061–2067. 9230382 10.1128/jcm.35.8.2061-2067.1997 PMC229903

[B17] Falco I. Verdegue M. Aznar R. Sanchez G. Randazzo W. (2019). Sanitizing food contact surfaces by the use of essential oils. *Innov. Food Sci. Emerg. Technol.* 51 220–228. 10.1016/j.ijfoodmicro.2015.08.021 26350124

[B18] Fasanmi G. O. Olukole S. G. Kehinde O. O. (2010). Microbial studies of table scrapings from meat stalls in Ibadan Metropolis, Nigeria: implications on meat hygiene. *Afr. J. Biotechnol.* 9 3158–3162.

[B19] Flemming H. C. Leis A. (2001). “Extracellular polymeric substances - the construction material of biofilms,” in *Proceedings of the 1st IWA International Conference on Microbial Extracellular Polymeric Substances, Water Science and Technology*, Vol. 43 Mulheim, Vii–Ix.

[B20] Gehrig M. Schnell C. Zurcher E. Kucera L. J. (2000). Hygienic aspects of wood and polyethylen cutting boards regarding food contaminations. A comparison. *Holz Als Roh-Und Werkstoff* 58 265–269.

[B21] Ghatak I. Chatterjee S. (2018). Urban street vending practices: an investigation of ethnic food safety knowledge, attitudes, and risks among untrained Chinese vendors in chinatown, Kolkata. *J. Ethnic Foods* 5 272–285.

[B22] Hausner M. Wuertz S. (1999). High rates of conjugation in bacterial biofilms as determined by quantitative in situ analysis. *Appl. Environ. Microbiol.* 65 3710–3713. 10427070 10.1128/aem.65.8.3710-3713.1999 PMC91555

[B23] Ismaïl R. Aviat F. Michel V. Le Bayon I. Gay-Perret P. Kutnik M. (2013). Methods for recovering microorganisms from solid surfaces used in the food industry: a review of the literature. *Int. J. Environ. Res. Public Health* 10 6169–6183. 10.3390/ijerph10116169 24240728 PMC3863893

[B24] Jing G. C. Sun Z. Wang H. L. Gong Y. H. Huang S. Ning K. (2017). Parallel-META 3: comprehensive taxonomical and functional analysis platform for efficient comparison of microbial communities. *Sci. Rep.* 7:40371. 10.1038/srep40371 28079128 PMC5227994

[B25] Kang K. Ni Y. Q. Li J. Imamovic L. Sarkar C. Kobler M. D. (2018). The environmental exposures and inner- and intercity traffic flows of the metro system may contribute to the skin microbiome and resistome. *Cell Rep.* 24 1190–1202.e5. 10.1016/j.celrep.2018.06.109 30067975

[B26] Khan H. A. Ahmad A. Mehboob R. (2015). Nosocomial infections and their control strategies. *Asian Pac. J. Trop. Biomed.* 5 509–514.

[B27] Khan H. A. Baig F. K. Mehboob R. (2017). Nosocomial infections: epidemiology, prevention, control and surveillance. *Asian Pac. J. Trop. Biomed.* 7 478–482. 10.1016/j.apjtb.2017.01.019

[B28] Kim S. Y. Li T. Heo J. Y. Bae Y. M. Hwang I. K. Lee S. Y. (2012). Efficacies of cleaning methods for decontaminating *Vibrio parahaemolyticus* on the surfaces of cutting boards cross-contaminated from grated fish fillet. *J. Food Saf.* 32 459–466. 10.1111/jfs.12005

[B29] Klontz K. C. Timbo B. Fein S. Levy A. (1995). Prevalence of selected food-consumption and preparation behaviors associated with increased risks of food-borne disease. *J. Food Prot.* 58 927–930. 10.4315/0362-028X-58.8.927 31137398

[B30] Kotzekidou P. (2016). “Factors influencing microbial safety of ready-to-eat foods,” in *Food Hygiene and Toxicology in Ready-to-Eat Foods*, ed. Kotzekidou P. (Cambridge, MA: Academic Press), 33–50. 10.1016/b978-0-12-801916-0.00003-0

[B31] Kövesdi V. Stercz B. Ongrádi J. (2016). Kurthia gibsonii as a sexually transmitted zoonosis: from a neglected condition during World War II to a recent warning for sexually transmitted disease units. *Indian J. Sex. Transm. Dis.* 37 68–71. 10.4103/0253-7184.180296 27190416 PMC4857686

[B32] Mahajan R. Garg S. Sharma P. B. (2014). Global food safety: determinants are Codex standards and WTO’s SPS food safety regulations. *J. Adv. Manag. Res.* 11 176–191. 10.1108/jamr-01-2013-0007

[B33] Minh N. P. (2017). Food safety knowledge and hygiene practice of street vendors in mekong river delta region. *Int. J. Appl. Eng. Res.* 12 15292–15297.

[B34] Moremi N. Claus H. Mshana S. E. (2016). Antimicrobial resistance pattern: a report of microbiological cultures at a tertiary hospital in Tanzania. *BMC Infect. Dis.* 16:756. 10.1186/s12879-016-2082-1 27964724 PMC5154146

[B35] Navas M. E. Hall G. El Bejjani D. (2013). A case of endocarditis caused by *Lactococcus garvieae* and suggested methods for identification. *J. Clin. Microbiol.* 51 1990–1992. 10.1128/JCM.03400-12 23554190 PMC3716042

[B36] Otoole D. K. (1995). Microbiological quality of pork meat from local Hong-Kong markets. *World J. Microbiol. Biotechnol.* 11 699–702. 10.1007/BF00361025 24415030

[B37] Podschun R. Ullmann U. (1998). *Klebsiella* spp. as nosocomial pathogens: epidemiology, taxonomy, typing methods, and pathogenicity factors. *Clin. Microbiol. Rev.* 11 589–603. 10.1128/cmr.11.4.589 9767057 PMC88898

[B38] Prechter S. Betz M. Cerny G. Wegener G. Windeisen E. (2002). Hygiene aspects of wooden resp plastic cutting boards. *Holz Als Roh-Und Werkstoff* 60 239–248.

[B39] Rutgersson C. Fick J. Marathe N. Kristiansson E. Janzon A. Angelin M. (2014). Fluoroquinolones and qnr genes in sediment, water, soil, and human fecal flora in an environment polluted by manufacturing discharges. *Environ. Sci. Technol.* 48 7825–7832. 10.1021/es501452a 24988042

[B40] Sorensen S. J. Bailey M. Hansen L. H. Kroer N. Wuertz S. (2005). Studying plasmid horizontal transfer in situ: a critical review. *Nat. Rev. Microbiol.* 3 700–710. 10.1038/nrmicro1232 16138098

[B41] Stackebrandt E. Liesack W. (1993). “Nucleic acids and classification,” in *Handbook of New Bacterial Systematics*, eds Goodfellow M. O’donnell A. G. (London: Academic Press), 152–189.

[B42] Stewart P. S. (2001). Multicellular resistance: biofilms. *Trends Microbiol.* 9 204–204. 11393180 10.1016/s0966-842x(01)01983-7

[B43] Stewart P. S. (2003). Diffusion in biofilms. *J. Bacteriol.* 185 1485–1491. 10.1128/jb.185.5.1485-1491.2003 12591863 PMC148055

[B44] Szczepanowski R. Linke B. Krahn I. Gartemann K. H. Gutzkow T. Eichler W. (2009). Detection of 140 clinically relevant antibiotic-resistance genes in the plasmid metagenome of wastewater treatment plant bacteria showing reduced susceptibility to selected antibiotics. *Microbiol. Sgm* 155 2306–2319. 10.1099/mic.0.028233-0 19389756

[B45] Thanigaivel G. Anandhan A. (2015). Isolation and characterization of microorganisms from raw meat obtained from different market places in and around Chennai. *J. Pharm. Chem. Biol. Sci.* 3 295–301.

[B46] Tran B. X. Do H. T. Nguyen L. T. Boggiano V. Le H. T. Thi Le X. T. (2018). Evaluating food safety knowledge and practices of food processors and sellers working in food facilities in Hanoi, Vietnam. *J. Food Prot.* 81 646–652. 10.4315/0362-028X.JFP-17-161 29543525

[B47] Webber M. A. Piddock L. J. V. (2003). The importance of efflux pumps in bacterial antibiotic resistance. *J. Antimicrob. Chemother.* 51 9–11. 10.1093/jac/dkg050 12493781

[B48] Weisburg W. G. Barns S. M. Pelletier D. A. Lane D. J. (1991). 16s Ribosomal DNA amplification for phylogenetic study. *J. Bacteriol.* 173 697–703. 10.1128/jb.173.2.697-703.1991 1987160 PMC207061

[B49] Wu X. Ye Y. Hu D. Liu Z. Cao J. (2014). Food safety assurance systems in Hong Kong. *Food Control* 37 141–145. 10.1016/j.foodcont.2013.09.025

